# Predictors of Functional Outcome in Patients With Bipolar Disorder: Effects of Cognitive Psychoeducational Group Therapy After 12 Months

**DOI:** 10.3389/fpsyt.2020.530026

**Published:** 2020-11-23

**Authors:** Gabriele Sachs, Andrea Berg, Reinhold Jagsch, Gerhard Lenz, Andreas Erfurth

**Affiliations:** ^1^Department of Psychiatry and Psychotherapy, Medical University of Vienna, Vienna, Austria; ^2^Department for Clinical and Health Psychology, University of Vienna, Vienna, Austria; ^3^First Department of Psychiatry and Psychotherapeutic Medicine, Klinik Hietzing, Vienna, Austria

**Keywords:** bipolar disorder, cognitive function, psychosocial functioning, occupational impairment, symptom severity, recurrence, cognitive psychoeducational group therapy

## Abstract

**Background:** Cognitive deficits are known as a core feature in bipolar disorder. Persisting neurocognitive impairment is associated with low psychosocial functioning. The aim of this study was to identify potential cognitive, clinical and treatment-dependent predictors for functional impairment, symptom severity and early recurrence in bipolar patients, as well as to analyze neurocognitive performance compared to healthy controls.

**Methods:** Forty three remitted bipolar patients and 40 healthy controls were assessed with a neurocognitive battery testing specifically attention, memory, verbal fluency and executive functions. In a randomized controlled trial, remitted patients were assigned to two treatment conditions as add-on to state-of-the-art pharmacotherapy: cognitive psychoeducational group therapy over 14 weeks or treatment-as-usual. At 12 months after therapy, functional impairment and severity of symptoms were assessed.

**Results:** Compared to healthy controls, bipolar patients showed lower performance in executive function (perseverative errors *p* < 0.01, categories correct *p* < 0.001), sustained attention (total hits *p* < 0.001), verbal learning (delayed recall *p* < 0.001) and verbal fluency (*p*-words *p* < 0.002). Cognitive psychoeducational group therapy and attention predicted occupational functioning with a hit ratio of 87.5%. Verbal memory recall was found to be a predictor for symptom severity (hit ratio 86.8%). Recurrence in the follow-up period was predicted by premorbid IQ and by years of education (hit ratio 77.8%).

**Limitations:** Limitations of the present study result mainly from a small sample size. The extent of cognitive impairment appears to impact occupational disability, clinical outcome as well as recurrence rate. This result must be interpreted with caution because statistical analysis failed to show higher significance.

**Conclusions:** Bipolar patients benefit from cognitive psychoeducational group therapy in the domain of occupational life. Deficits in sustained attention have an impact on occupational impairment. Implications for treatment strategies are discussed. Further evaluation in larger studies is needed.

## Introduction

Growing evidence suggests a considerable gap between syndromal and functional recovery among bipolar patients (1). Bipolar disorder (BD) is a severe mental illness; the course of the disease and the clinical outcome can be quite diverse. Treatment of BD often requires long-term medication and subsequently treatment adherence. There is a close relationship between treatment adherence and neurocognitive impairment (2). Neurocognitive deficits are known as a core feature in BD (3), including euthymic BD (4–6), subgroups (7) and are present during all stages (8) with a heterogeneous profile (4). Bipolar patients show poor performance in most cognitive domains, in processing speed, attention, verbal memory and executive functioning; in particular, context processing performance and associative learning are impaired (9, 10). Cognitive deficits are similar in bipolar I and bipolar II patients (11). Bipolar patients with psychosis exhibited more poorly on memory and executive function and had lower psychosocial functioning predicting limited recovery (12). There is not enough evidence so far for cognitive deficits being progressive in BD. A meta-analytic study did not support the hypothesis of a progressive decline of cognitive deficits (13). Cognitive dysfunction seems to be stable over time, only dysfunction in verbal recall was found to show a progressive course in a 5-year follow-up study (14). Medial temporal dysfunction (15) and reduced white matter integrity (16) have been suggested to be involved with verbal memory impairment early in the course of bipolar I disorder.

Psychosocial dysfunction may differ when patients with first- and multiple-episode BD are compared. Repeated episodes may contribute to higher impairment in multiple areas of psychosocial functioning. Clinical factors such as depressive symptoms seem to have a negative impact on functioning (17). Persisting neurocognitive impairments are also found to be associated with low psychosocial functioning (18–20), specifically with significant impairment in work, family and social life, beyond the acute phases of the illness and depending on the patient's own evaluations of their subjective functioning (21).

There is a need for further knowledge regarding predictors of the course of the disorder. Previous number of mixed episodes, subdepressive symptomatology, number of hospitalizations and older age were found to predict functional outcome. Bonnín et al. (22) demonstrated that subdepressive symptomatology together with neurocognitive impairments related to verbal memory and executive functions are predictor variables of long-term functional outcome in BD. The number of hospitalizations for depressive episodes and illness duration were associated with a reduction in occupational functioning in patients suffering from BD (23). Ryan et al. (24) examined impact of cognition on work status and underemployment in euthymic BD patients. Patients with BD who have better cognitive functioning are more likely to be employed. After accounting for number of mood episodes patients with BD who are unemployed/unable to work have greater difficulties processing emotional information and on executive tasks comprising a shifting or interference resolution component compared to BD patients who are employed. Bonnín et al. (25) examined predictors of functional outcome after a manic episode and showed that number of past depressive episodes, psychotic symptoms at index episode, and Body Mass Index (BMI) predict worse outcome after 6 months follow-up.

There is evidence that maintenance pharmacotherapy in conjunction with psychological interventions can improve the outcome (26). Adjunctive psychosocial interventions (27–36), including web-based and mobile approaches have been suggested to enhance symptomatic and functional outcome (37–43) in patients with BD -comprising adolescents (44)-, as well as in patients with subthreshold manifestations such as cyclothymic disorder (45). Presence of mixed episodes, full medication adherence and therapeutic blood levels of mood stabilizers were found predictive to attend a psychoeducation program (46). In a study with 55 patients with BD I and II in remission, 16-session psychoeducation seemed to be ineffective to prevent mood episodes or improve functioning (47). Functional remediation, a novel group intervention, showed efficacy in improving the functional outcome of a sample of euthymic bipolar patients as compared with treatment as usual (48, 49). Improvement in psychosocial functioning after functional remediation has been shown to be maintained after 1-year follow-up (50).

Clinical features and cognition related to functional outcome may have important effects on the overall therapeutic outcome. There is little evidence which clinical and neurocognitive variables would best predict the functional outcome in BD patients.

Aim of the present study was to identify potential cognitive, clinical and treatment-dependent predictors on functional impairment, symptom severity and early recurrence in patients with BD. As an additional predictor, the impact of cognitive psychoeducational group therapy [CPEGT, (51)] on psychosocial function was explored. Furthermore, it was assessed whether bipolar patients and healthy controls differ regarding neurocognitive performance in executive functions and sustained attention.

## Methods

### Participants

Remitted bipolar patients were recruited consecutively from psychiatric outpatient and inpatient units at the Department of Psychiatry and Psychotherapy, Medical University of Vienna, Austria (*N* = 43) with the following inclusion criteria: age between 18 and 65 years, diagnosis of bipolar I or II disorder according to DSM-IV TR criteria using the Structured Clinical Interview for DSM-IV (SCID) (52) and the MINI [Neuropsychiatric Interview, (53)], at least two episodes in the last 3 years or three episodes in the last 5 years, ongoing medication with mood stabilizers and remission. Remission was defined by the following scores: Beck Depression Rating Scale <18 [BDI, (54)], Manie-Selbstbeurteilungsskala <14 [MSS: German version of the Self-Report Manic Inventory, SRMI, (55)], Bech Rafaelsen Mania Scale <9, [BRMAS, (56)], Bech Rafaelsen Melancholia Scale <26 [BRMES, (57)].

Exclusion criteria were intellectual disability, history of substance abuse, neurological disease, as well as any medical condition that could affect neuropsychological performance. Patients were also excluded if they had participated in any structured psychological intervention, such as psychoeducation or cognitive remediation, within the past 2 years.

To assess neurocognitive performance in bipolar patients in comparison to healthy volunteers, 40 controls matched by age, gender and years of education were included into the study. Using the MINI it was ensured that the controls had no antecedent of neurological disease, no current diagnosis or history of psychiatric illness, current or previous alcohol/substance dependence or abuse, and that they were not taking psychotropic medication.

All participants, patients and controls, were fluent in German. The study was conducted in accordance with the ethical principles of the Declaration of Helsinki and Good Clinical Practice. The study protocol was approved by the Vienna University Hospital Ethics Committee. All subjects received extensive information about the study and gave written informed consent for their participation before they were enrolled in the study.

### Assessments

Demographical and clinical assessment: In addition to the SCID and MINI, all patients were evaluated with BDI, MSS, BRMES, and BRMAS.

Demographical and clinical information were obtained from clinical charts and patient interviews (age, gender, years of education, duration of illness, bipolar subtype, number of manic, hypomanic and depressive episodes, history of psychosis, current medication). The severity of symptoms was assessed using the Clinical Global Impression scale [CGI, (58)].

Functional impairment in areas of vocational, social and family was rated applying the Sheehan Disability Scale [SDS, (59)].

Neuropsychological assessment: At baseline bipolar patients completed a neurocognitive test battery including different tasks divided into cognitive domains: estimated premorbid IQ, using a German multiple choice vocabulary test, the Mehrfachwahl-Wortschatz-Test [MWT, (60)]; attention/vigilance, which was evaluated by the Continuous Performance Test, Identical Pairs [CPT-IP, (61)]; executive function (set shifting, planning and response inhibition) using the Computerized Wisconsin Card Sorting Test [WCST, (62)]; verbal learning and memory which were assessed by the Verbal Learning and Memory Test [VLMT, (63)] and verbal fluency using the Regensburger Word Fluency Test [RWT, (64)].

Reassessment: Patients underwent clinical and functional reassessment after completion of the intervention (14 weeks after baseline evaluation). Furthermore, a follow-up assessment was performed after 12 months.

At 12 months functional impairment in areas of vocational, social and family life was assessed applying the SDS, severity of symptoms was rated using the CGI. The recurrence rate was established. Recurrence was diagnosed when full DSM-IV TR criteria for a new affective episode were met; adherence toward psychopharmacology was measured using the Medication Compliance Questionnaire [MCQ, (65)].

### Interventions

Of the 43 bipolar patients, 19 (12 female) were randomly assigned to the intervention group and 24 to the treatment as usual (14 female). There were no significant differences between the groups with regard to age, gender, BD I/BD II, rapid cycling, duration of illness, number of episodes, education, being in a job or current medication (choice of mood stabilizer, taking an antipsychotic). All 43 patients received state-of-the-art pharmacotherapy and completed the study.

Patients were randomized to either cognitive psychoeducational group therapy (CPEGT) (51, 66) over 14 weeks or to treatment as usual (TAU information group).

CPEGT consisted of 14 sessions; the duration of each session was 90 min. The contents of CPEGT were distributed in the following way: session 1–introduction and overview, session 2–explanatory models of bipolar disorders, session 3–pharmacotherapy, session 4–side effects of medications, session 5–depression I–symptoms and coping strategies, session 6–depression II—increase of pleasant activities, session 7–depression III—modification of depressive cognitive patterns, session 8–depression IV–relapse prevention, session 9–mania I–symptoms and coping strategies, session 10–mania II–relapse prevention, session 11–healthy lifestyle I–regular rhythm of life without alcohol and drugs, session 12–healthy lifestyle II—the “Life Chart Method,” session 13–healthy lifestyle III–goal attainment and communication strategies, session 14–review.

Additionally, between two and four sessions were performed with relatives.

Patients in the control group also had regular group sessions. The thematic focus was on reading a book about BD [“Sturzfliegen” by Vasak and Katschnig, (67)]. In addition, three questions and answers sessions were provided. Booster sessions were offered after 6 and 9 months for both treatment and control group.

### Statistical Analysis

Data were analyzed using the Statistical Package of Social Sciences (SPSS Inc. 19^th^ Version, Chicago, IL, USA). Differences in demographic characteristics and neurocognitive measures between patients with bipolar disorder and controls were studied using independent *t*-test for continuous variables. Chi–square was used to examine categorial data. Pearson correlations were calculated to identify which demographic, clinical and neurocognitive variables were linked to functional impairment (three domains assessed by the SDS), symptom severity (CGI) and recurrence rate (number of episodes in the follow-up period of 12 months). The demographic variables were age, gender, years of education. The clinical variables were CPEGT, duration of illness, BDI, MSS, number of affective episodes in the 12 months prior to the start of the study, history of psychosis, current medication. The neuropsychological variables were premorbid IQ (MWT), attention/vigilance (CPT-IP, CPT –IP total hits, total false), WCST categories (categories correct, perseverative errors, verbal learning and memory (VLMT, immediate recall, delayed recall) and verbal fluency (RWT, *p*-words, *s*-words).

All significant variables (demographic, clinical and neurocognitive) that were found to correlate with functioning, CGI and recurrence rate were introduced in a five backwards stepwise logistic regression analyses. Additional variables from the literature, including correlations between clinical, neurocognitive measures and functional outcome, were considered (23). Backward (Wald) logistic regressions were used to predict functioning, CGI and recurrence rate and estimate percentage variance accounted for by variables of interest.

Level of significance was set at ≤0.05.

## Results

### Demographic and Clinical Characteristics

Thirty three patients with bipolar I disorder and 10 patients with bipolar II disorder were included; of the 43 bipolar patients seven were rapid cyclers. The age (mean ± SD) was 42.2 ± 10.9 years, the years of education 16.0 ± 4.0. The average duration of illness was 15.3 ± 10.3 years; the number of affective episodes in the 12 months prior to the start of the study was 3.4 ± 2.4 [0–8]. Fifteen patients (34.9%) had a history of psychotic symptoms, 26 (60.5%) were female. All 43 patients received at least one mood stabilizer (lamotrigine 17, sodium valproate/valproic acid 16, lithium carbonate 11, carbamazepine 3); additional medications were atypical antipsychotics (22 patients), antidepressants (10) and hypnotic drugs (benzodiazepines/zolpidem: 5). For an overview of all demographic, clinical and neurocognitive characteristics see Table 1.

**Table 1:** Demographic, clinical and neurocognitive characteristics of patients with BD at baseline (*n* = 43).

**Variables**	**Mean**	**SD**
Age, years	42.2	10.9
Education, years	16.0	4.0
Duration of illness, years	15.3	10.3
Number of affective episodes in the 12 months prior to the start of the study	3.4	2.4
	***N***	**%**
Female	26	60.5
Male	17	39.5
History of psychotic symptoms	15	34.9
**Medication**		
Lithium carbonate	11	25.6
Sodium valproate/valproic acid	16	37.2
Lamotrigine	17	39.5
Carbamazepine	3	7.0
Atypical antipsychotics	22	51.1
Antidepressants (SSRI)	10	23.3
Hypnotic drugs (benzodiazepines/zolpidem)	5	11.6
**Neurocognition**	**Mean**	**SD**
MWT	30.5	4.2
WCST total correct	68.3	13.9
WCST perseverative errors	19.2	18.7
CPT-IP Total hits	0.47	0.2
CPT-IP Total false	0.16	0.1
VMLT immediate recall	9.95	2.8
VMLT delayed recall	7.04	2.0
RWT *p*-words	12.60	4.8
RWT *s*-words	21.11	3.8

*MWT, Mehrfach-Wortschatz-Test: assessment of premorbid intelligence; WCST, Wisconsin Card sorting test; CPT-IP, Continuous Performance Test, identical pairs; VMLT, Verbal Learning and Memory Test; RWT, Regensburger Word Fluency Test*.

### Neurocognitive Performance in Bipolar Patients

#### Comparison With Healthy Controls

Neurocognitive performance was compared to 40 healthy controls matched by age, sex and years of education. Compared to healthy controls, patients with BD showed lower performance in executive functions as measured by the WCST (perseverative errors *p* ≤ 0.01, categories correct *p* ≤ 0.001), in sustained attention (CPT-IP total hits *p* ≤ 0.001), in verbal learning (VLMT delayed recall *p* ≤ 0.001) and verbal fluency (RWT *p*-words *p* ≤ 0.002).

#### Pearson Correlations of Demographic, Clinical, and Neurocognitive Characteristics of Patients With BD (Table 2)

CPT-IP false hits (*r* = 0.36, *p* = 0.023) and WCST total correct (*r* = 0.41; *p* = 0.009) correlated with number of affective episodes in the 12 months prior to the start of the study.

**Table 2:** Pearson correlations of demographic, clinical and neurocognitive characteristics of patients with BD at baseline (*n* = 43).

	**MWT**	**CPT-IP hits**	**CPT-IP false**	**WCST total**	**WCST persev**.	**VMLT immediate**	**VMLT delay**	**RWT *p*-words**	**RWT *s*-words**
Gender	−0.12	−0.01	0.06	0.08	0.15	0.22	0.11	0.22	−0.10
Age	0.30^*^	−0.17^*^	−0.07	−0.00	0.20^*^	−0.23	−0.33^*^	0.18	0.10
Years of education	0.24^*^	−0.01	−0.14	0.01	−0.13	0.01	−0.18	0.16	0.22
Illness duration	0.25	−0.12	0.09	0.10	0.12	−0.13	−0.04	0.26	−0.10
Number of episodes	0.00	0.14	0.36^*^	0.41^**^	−0.17	−0.11	−0.04	0.01	−0.21
Psychosis	−0.34^*^	0.15	−0.10	0.15	−0.11	−0.02	0.11	−0.01	0.09
Atypical antipsychotics	−0.26	−0.17	−0.08	0.01	−0.04	−0.01	0.14	−0.05	0.03

* p ≤ 0.05,

** *p ≤ 0.01*.

*MWT, Mehrfach-Wortschatz-Test: assessment of premorbid intelligence; CPT-IP hits, continuous performance test total hits; CPT-IP false, continuous performance test total false; WCST total, Wisconsin Card sorting test, total correct; WCST persev., Wisconsin Card sorting test, perseverative errors; VMLT immediate, Verbal Learning and Memory Test, immediate recall; VMLT delay, Verbal Learning and Memory Test, delayed recall; RWT pwords, Regensburger Word Fluency Test p-words; RWT s-words, Regensburger Word Fluency Test s-words; Number of episodes, number of affective episodes in the 12 months prior to the start of the study*.

### Effects of Therapy

#### Effects of Therapy on Number of Episodes

As previously communicated (68, 69), after 14 weeks of therapy an improvement in illness concepts and adherence was observed in both the CPEGT group and the TAU group; after further 12 months the number of manic episodes was significantly decreased in both groups as compared to the 12 months before the intervention. A significant reduction of the number of depressive episodes was observed after CPEGT; after TAU the number of depressive episodes remained unchanged.

#### Functional Impairment, Severity of Symptoms, and Early Recurrence: Pearson Correlations

SDS score impairment in areas of vocational functioning correlated with CPEGT (*r* = 0.381; *p* = 012) and CPT-IP (false *r* = −0.411; *p* = 0.006). Scores in CGI correlated with verbal learning immediate recall (*r* = 0.338, *p* = 0.038) and verbal learning delayed recall (*r* = 0.336, *p* = 0.039). Finally, recurrence was found to correlate with verbal learning immediate recall (*r* = 0.381, *p* = 0.018) and verbal learning delayed recall (*r* = 0.397, *p* = 0.014).

#### Regression Analyses

As shown in Table 3, CPEGT and CPT-IP (total false) predicted the amount of occupational impairment (hit ratio of 87.5%). VLMT delayed recall was found to be a predictor for symptom severity as measured by CGI (hit ratio 86.8%). Recurrence in the follow-up period of 12 months was predicted by MWT and years of education (hit ratio 77.8%).

**Table 3:** Functional impairment, severity of symptoms and early recurrence: results of regression analyses.

	**Regression coefficient B**	**Wald**	**Exp(B)**	***p***
Occupational impairment (SDS)				
Cognitive psychoeducational group therapy (CPEGT)	−2.30	6.68	0.10	0.010^**^
CPT-IP total false	−11.73	5.45	0.00	0.020^*^
CGI				
Atypical antipsychotics	−1.82	3.03	0.16	0.082
VLMT delayed recall	0.48	4.24	1.62	0.039^*^
Early recurrence				
VLMT delayed recall	0.34	3.64	1.41	0.056
MWT	0.36	4.93	1.43	0.026^*^
Years of education	−0.38	5.68	0.69	0.017^*^

*Significance level was set at

* p ≤ 0.05,

** *p ≤ 0.01*.

*SDS, Sheehan Disability Scale at 12-month follow-up; CPT-IP total false, Continuous Performance Test total false at baseline; CGI, Clinical Global Impression scale at 12-month follow-up; early recurrence, occurrence of an affective episode within the 12-month followup period; VLMT delayed recall, Verbal Learning and Memory Test, delayed recall at baseline; MWT, Mehrfach-Wortschatz-Test: assessment of premorbid intelligence at baseline*.

Of these predictors reaching the previously defined significance level of *p* ≤ 0.05, CPEGT (with regard to occupational impairment) was the only predictor reaching a significance level of *p* ≤ 0.01.

No significant predictors were found for social disability and family-life impairment.

## Discussion

Patients with BD are impaired in the domains of attention, verbal memory and executive functions (4). The results of our study confirm these findings showing specific deficits in sustained attention, executive function, verbal memory and also in verbal fluency. Remitted bipolar patients had clinically significant cognitive deficits compared to healthy controls. The patients in this study were characterized by a particularly long duration of illness (average of 15.3 years). Looking at the association between number of affective episodes in the 12 months prior to the start of the study and cognitive function a significant correlation was found between number of affective episodes and impairment in the domain of sustained attention (continuous performance test: total false). Unfortunately, due to the small sample size (*N* = 43), it was not possible to assess which type of past episodes (depressive, manic or mixed) led to the reduction of sustained attention in these remitted patients. Clearly, further research on neurocognitive dysfunction in rapid cycling bipolar disorder (70) is suggested by our finding. Data on cognitive impairment in bipolar patients might have important clinical and therapeutic implications and provide support for the necessity to use neurocognitive assessments in routine clinical examination in patients with BD (2–4, 71).

Cognitive deficits might be also an important factor for the success or failure of therapeutic interventions, both pharmacological and non-pharmacological. As cognitive deficits are a core feature in BD, recommendation should be given for specific cognitive interventions in subgroups of patients with clinically significant cognitive impairments. The effectiveness of different psychosocial interventions might be influenced by cognitive function: so far this has not been thoroughly studied to our knowledge. In this study bipolar patients were offered a cognitive psychoeducational group therapy program [CPEGT, (51)]. This study reports data from a logistic regression analysis looking at the impact of this training on functional outcome and recurrence rate. The sample in this study was characteristic in that patients showed a long duration of illness and a corresponding cognitive impairment.

Our study shows a significant impact of CPEGT on functional outcome in patients with BD; to a lesser degree also CPT-IP (false hits) predicted occupational impairment. These findings are clearly preliminary due to the small sample size, but CPEGT seems to be a useful additional therapy to medication to treat patients with long duration of illness. All patients completed the 14-week intervention group, which is consistent with a high acceptability of the treatment strategy. Our study on CPEGT supports the concept that specific psychosocial interventions targeting specific aspects of BD can be beneficial (72, 73).

Very little is known about the longitudinal course of cognitive deficits and the neuropsychological underpinnings of the dynamics of BD (74). Improving the knowledge on the development in time of cognitive dysfunction in BD [role of depressive episodes, role of manic episodes, role of mixed episodes, role of specific medications for better or for worse, role of cognitive remediation programs, role of inflammatory mediators (75), role of genetic factors (76, 77)] would contribute to identify targets for treatment, to determine possible subtypes of the disorder, and to develop better therapeutic strategies (78, 79). The metaanalytic study by Samamé et al. (13) showed that cognitive deficits remain stable after a follow-up period of 4.62 years. In a study of Demant et al. (80) no significant effect of shortterm cognitive remediation on cognitive dysfunction was found. In our study verbal learning memory deficits at baseline showed correlation with severity of symptoms after 12 months; we therefore suggest that specific intensive and individualized cognitive training may be indicated for this chronic subgroup of patients with BD.

## Limitations

Limitations of the present study clearly result from the small sample size. While the number of affective episodes in the 12 months prior to the start of the study was correlated to disturbances in sustained attention, it was not possible to assess which polarity of episodes was responsible for this effect.

## Conclusion

Compared to healthy controls bipolar patients show cognitive impairment.

The extent of cognitive impairment appears to impact occupational disability and clinical outcome.

Occupational impairment was reduced by cognitive psychoeducational group therapy (CPEGT).

## Data Availability Statement

The datasets generated for this study are available on request to the corresponding author.

## Ethics Statement

The studies involving human participants were reviewed and approved by Medical University of Vienna, Austria. The patients/participants provided their written informed consent to participate in this study.

## Author Contributions

GS wrote the manuscript, performed the statistics, coordinated, and performed the clinical study. AB contributed to the realization of group therapy and cognitive tests. RJ performed the statistics. GL planned, coordinated, and performed the clinical study. AE wrote the manuscript, reviewed the existing literature, and contributed to the recruitment of patients. All authors contributed to the article and approved the submitted version.

## Conflict of Interest

The authors declare that the research was conducted in the absence of any commercial or financial relationships that could be construed as a potential conflict of interest.
